# Effects of Physical Activity Programs on the Improvement of Dementia Symptom: A Meta-Analysis

**DOI:** 10.1155/2016/2920146

**Published:** 2016-10-13

**Authors:** Han Suk Lee, Sun Wook Park, Yoo Jung Park

**Affiliations:** ^1^Department of Physical Therapy, Faculty of Health Science, Eulji University, 212 Yangji-dong, Sujeong-gu, Seongnam-si, Gyeonggi-do 461-713, Republic of Korea; ^2^Department of Physical Therapy, Samsung Seoul Hospital, 81 Irwon-ro, Gangnam-gu, Seoul 06351, Republic of Korea

## Abstract

*Objective*. To confirm that physical activity program improves the symptoms of dementia and the most effective physical activity was selected to help establish exercise programs.* Methods*. Three databases, PubMed, Science Direct, and Willey online, were used to collect articles. The databases were published between January 2005 and December 2015. Keywords such as “dementia,” and “physical activity” were used in searching for papers. As a result, nine studies were selected in the second screening of the meta-analyses.* Results*. The improvement in the dementia symptom of physical capacity was 1.05 (high effect size, 95% CI: 0.03 to 0.73), ability of activity of daily living was 0.73 (slightly high effect size, 95% CI: 0.23 to 1.23), cognitive function was 0.46 (medium effect size, 95% CI: 0.26 to 0.66), and psychological state was 0.39 (lower than the medium effect size, 95% CI: 0.01 to 0.77).* Conclusion*. The physical activity for patients with dementia had an effect on the improvement of physical capacity and combined exercise was the most effective physical activity.

## 1. Introduction

Various physical activity programs have been developed for relieving symptoms of dementia [1–5]. They are effective for attention and executive functions; however, their effectiveness on memory is controversial [6]. Therefore, it is necessary to confirm how effective physical activity programs are for relieving the symptoms of dementia.

The physical activities that are recommended for patients with dementia include aerobic exercises, muscle-strengthening exercises, hydrotherapy, exercises involving music [7], and Taichi [8, 9]. Among these, aerobic exercises are highly recommended because they have many advantages such as reducing the hippocampal atrophy rate in patients with dementia [10]. However, walking is the physical activity that is recommended most often because it is easy and is associated with a low risk of falls. One to two hours of walking is effective for improving cognitive function [11]. In addition to walking, various other physical activities such as balance and muscle-strengthening exercises are combined in community exercise programs [12].

Nevertheless, the general population may be confused because of the diversity among the options about the effects of physical activity. Therefore, there is a need to suggest the most effective type of exercise and create guidelines for designing physical activity programs.

Some meta-analyses on the effects of exercise for subject with dementia were published. But, they included the articles that targeted not only elderly patients with dementia but also elderly patients with mild cognitive impairment for analyzing [9, 13, 14]. In addition, analyses of the articles that were recently published have not been sufficiently conducted. Therefore, we will analyze the articles that were focused on the elderly patients with dementia not elderly patients with mild cognitive impairment.

In this study, a meta-analysis of the physical activity programs for elderly patients with dementia was conducted. Our purpose was to confirm that physical activity program improves the symptoms of dementia and the most effective physical activity to help establish exercise programs.

## 2. Material and Method

### 2.1. Criteria for Selecting the Target Papers and the Collection Process

Three databases, PubMed (158), Science Direct (129), and Wiley online library (450), were used to collect papers on dementia treatment. The databases were published between January 2005 and December 2015. Keywords such as “dementia,” and “physical activity” were used in searching for papers. Outcomes were not included in the searching. Two independent reviewers screened the tile and abstracts of all studies to identify randomized controlled trials or duplicate study.

We considered the following primary outcomes: the cognitive function, physical capacity, ability of activity of daily living, and psychological state. The secondary outcomes were Geriatric Depression Scale, Wechsler Memory Scale, Rivermead Behavioural Memory Test, Symptom Check List Anxiety, cardiopulmonary function, muscle strength, endurance, flexibility, activity of daily living disability score, 6 M walk, Neuropsychiatric Inventory Total Score, Montgomery-Asberg Depression Rating Scare, Mini Mental State Examination, Alzheimer's Disease Assessment Scale, Functional Reach Test, Time Up and Go Test, sit to stand test, Barthel Index, Instrumental ADL, and Cambridge Neuropsychological Test Automated Battery.

### 2.2. Data Extraction

Analyses were conducted through the following process. Based on the agreements among the coauthors of this study, data coding was conducted with items comprising the author's name, year of publication, publishing type, study model, study participants, genders of the participants, survey tool used, program type, main residence of the participants, and program effects. The coding was conducted by one graduate school student and one meta-analysis specialist. When a discrepancy in coding occurred, the opinions of the coauthors were reflected to address it. Accordingly, the reliability and consistency between the people who were coding were not calculated together.

### 2.3. Data Synthesis and Analysis

Analysis of the tables and calculation pertaining to effect sizes and* Q*-values, which were used for the meta-analyses, were conducted using CMA software, which is specifically designed for meta-analyses. Cohen and Wolf's criteria were used to interpret the effect size. A 0.2 or smaller effect size implied a small effect, 0.5 implied a medium effect, and 0.8 or greater implied a large effect [15]. As a result of the homogeneity test, the* Q*-value was 102.17, df was 8, and *I* ^2^ was 92.17, showing diversity (Table 1). Thus, random effect models were used for effect size measurement. The effect sizes were compared using the categorical variables that reflected the study features. The significance was accepted for values of *p* < 0.05.

## 3. Results

### 3.1. Description of Studies

**Figure 1:**
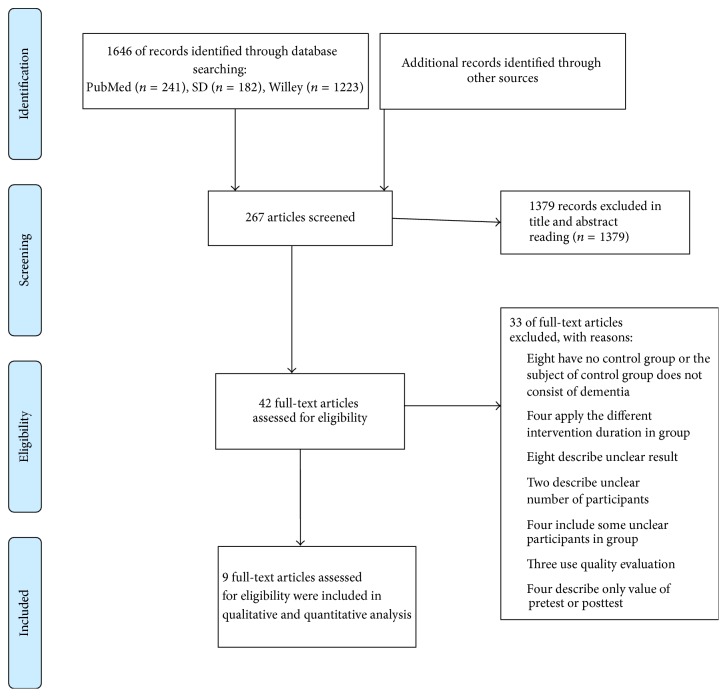
Flow diagram of studies included.

1646 articles were included for searching strategy. From these articles, 1379 were excluded for a detailed analysis and 267 articles were screened. After full-text reading, thirty-three of full-text articles were excluded for the following reasons. First, eight articles included the inadequate control group (there was not control group or the subject of control group is not dementia patient). Second, four articles were consisted of the different intervention duration between groups. Third, eight articles described unclear result that did not provide enough statistical data pertaining to meta-analyses. Forth, two articles unclearly described the number of participants. Fifth, four articles included the unclear participants that mixed the subject with dementia and MCI. Sixth, three articles used quality evaluation. Seventh, four articles described only value of pretest or posttest as results. Finally, nine studies were selected in the second screening of the meta-analyses.  Figure 1 shows the flow diagram of the studies according to PRISMA flow diagram and Table 2 summarized their characteristics.

### 3.2. Publication Bias Verification

To ensure the validity of the meta-analysis results, the publication bias was verified. No publication bias was confirmed (Table 3). When sensitivity analysis was conducted using the “trim and fill” method of Duval and Tweedie [21], the corrected and observed values were identical.

### 3.3. Effect Size according to Improvement of the Dementia Symptom

The improvement in the dementia symptom of physical capacity was 1.05 (high effect size, 95% CI: 0.03 to 0.73), activities of daily living were 0.73 (slightly high effect size, 95% CI: 0.23 to 1.23), cognitive function was 0.46 (medium effect size, 95% CI: 0.26 to 0.66), and psychological state was 0.39 (lower than the medium effect size, 95% CI: 0.01 to 0.77) (Table 4).

### 3.4. Effect Size according to the Type of Physical Activity Program

According to type of physical activity, the effect size of combined exercise program was 1.17 (high effect size, 95% CI: 0.86 to 1.47). The walking program was 0.46 (medium effect size, 95% CI: 0.18 to 0.74). Special physical activity program was 0.44 (medium effect size, 95% CI: 0.16 to 0.72). Walking program with other activities was 0.41 (medium effect size, 95% CI: 0.16 to 0.67) (Table 5).

## 4. Discussion

In this study, various physical activity programs for elderly patients with dementia were analyzed using the papers published since 2005 to find out the programs that are effective for relieving dementia symptoms and to suggest the most effective program.

The physical activity programs were very effective in improving the physical capability and ADL of patients with dementia but showed a small effect size in cognitive function or psychological states.

With aging, the frequency of occurrence of depression is increasing in the women with low level of physical activity [22]. And, higher social activity is related to a lower risk of depression [22]. This means that lower physical activity induces lower physical function and ability of activity of daily living and affects negative effect in the depression. Therefore, various programs have been developed to improve the depression in the elderly. However, the opinions of authors about the relationship between the physical activity and depression in elderly have conflicted [23].

In this study, the assessment tools of depression were Geriatric Depression Scale and Montgomery-Asberg Depression Rating Scale that lower score indicates normal state. We found that physical activity induces lower score of depression. This result can interpret positive effect in depression even though the effect size was small.

In Heyn et al.'s meta-analysis study [13] on the effects of physical activities, physical activities showed a large effect on the health-related physical fitness components but a comparatively small effect on the cognitive aspects, corresponding with the results of this study. In Farina et al.'s meta-analysis [24], physical activities were effective in the cognitive aspects; however, that study design was too diverse. In this study, the papers that were analyzed showed diverse designs, and, as such, the program design differences, according to the subjects and program features, should be reviewed in future study.

In terms of the effect size by the type of physical activity, the combined exercise method showed the largest size while the walking-alone method showed the smallest size among all of the methods. These results are similar to those of Scherder's meta-analysis [25], which confirmed a small effect size of walking activity on executive functions in cognitively normal elderly people with sedentary lifestyles.

Many studies recommend walking exercises because they are effective for cognition [1, 2, 4]. In particular, Winchester et al. [11] suggested the appropriate walking time. When Alzheimer's Disease (AD) patients in the study engaged in walking exercise for two hours or more every week, their Mini Mental State Examination (MMSE) scores improved. Nevertheless, these studies did not select the proper control groups that could benefit from engaging in physical activities but selected the groups that did simple ADL, such as those with a sedentary lifestyle who engaged in personal communication, who paid social visits (students), and who engaged in usual activities. In this study, the effect size of walking was relatively small because walking program was compared with combined exercise program or walking program with other activities. In other words, the control group of walking program was special. As suggested in the previous relevant studies, walking can be effective for cognitive improvement, but we cannot conclude it is not the most effective exercise compared with combined exercise program or walking program with other activities.

The World Health Organization (WHO) [26] recommends aerobic exercise and integrated exercises, consisting of aerobic and muscle-strengthening exercises, to prevent reduction in cognitive function in normal elderly people. In their study, Ukropcova et al. [27] reported that integrated aerobic-muscular exercise improved cognitive and muscular functions in patients with mild cognitive impairment (MCI). Ngandu et al. [12] developed the FINGER program for elderly people at risk of developing dementia, which includes physical exercise, cognitive training, diet control, and social training, that is, multidomain interventions that could improve or maintain the cognitive functions of such elderly patients with dementia. Among them, the physical exercise training program included aerobic, strengthening, and postural balance exercises. In this study, combined exercise program for patients with dementia showed positive effects and these results corresponded with those of the previous relevant studies. Therefore, combined exercise can be suggested as a treatment program guideline for patients with dementia.

Brain-derived neurotropic factor (BDNF) increases with aerobic and strengthening exercises. BDNF is known to help in supporting the survival of existing neurons and encouraging the growth of new neurons [28]. It is considered to be one of the most important factors in learning, memory, and higher thinking [29] and is reduced in elderly patients with MCI, Parkinson's disease, or dementias [30, 31]. Nascimento et al. [32] reported that the BDNF genotype seems to modulate the effects of physical exercise on BDNF secretion; however, it did not affect cognition and inflammatory pathways in elderly patients with MCI after a 16-week multimodal physical exercise program. de Melo Coelho et al. [33] suggested that moderate-intensity exercise seems to be more effective in promoting the increase in peripheral levels of BDNF in the elderly.

Boyle et al. [34] found that the muscle strength of the arms and legs was associated with a decreased risk of MCI and AD after a follow-up period of 3.6 years in 900 community-based elderly patients with dementia. Alfaro-Acha et al. [35] found that handgrip strength was related to decline in cognitive function over a 7-year period in older Mexican Americans. They reported that subjects with higher handgrip strengths had higher levels of cognitive function. Both researchers demonstrated the association of muscle strength and cognitive function. The reason for the association between muscle strength and AD is not clear. However, Alfaro-Acha et al. [35] suggest that muscle strength may be affected by the integrity of nervous system activity and that the relationship of pathogenic factors, such as high oxidative stress, high inflammatory markers, and low sex steroid levels, might be responsible for both muscle loss and cognitive decline.

Marosi et al. [36] found that long term physical exercise decreases the level of reactive oxygen species and protein carbonyls in the hippocampus that protect neurons against oxidative stress of aging rats. The physical activity may protect the hippocampal area that is related to the retrieval of episodic memories [37].

Lista and Sorrentino [38] suggest the biological mechanism to explain the role of physical activity in enhancement of cognition. They try to combine the interactions between different system (e.g., nervous, cardiovascular, and endocrinological), between molecular and cellular mechanisms, and, finally, between different molecules to explain the mechanism and suggest the supramolecular mechanisms (e.g., neurogenesis, synaptogenesis, and angiogenesis) but still would not explain the relationship between exercise and genetic polymorphisms.

Additionally, physical activity can affect the apolipoprotein E (*APOE*) genotypes which were divided as* APOEε*4 carriers (*ε*2/*ε*4, *ε*3/*ε*4, and *ε*4/*ε*4 genotypes) and noncarriers (*ε*2/*ε*2, *ε*2/*ε*3, and *ε*3/*ε*3 genotypes) because* APOE* can affect cognitive decline [38]. Bretsky et al. [39] and Podewils et al. [40] suggested that* APOEε*4 allele noncarriers were associated with physical activity. However, the mechanism is not clear.

According to earlier studies, aerobic exercise and strength exercise can affect the cognitive decline that is related to dementia, even though the mechanism remains unclear. Therefore, combined exercise, which consists of aerobic exercise and strength exercise, might be beneficial for elderly people with dementia. The outcomes of this study can serve as a scientific groundwork for the claim that integrated exercises, combining various exercises, are the most appropriate exercises among the various types of known exercise. Therefore, combined exercise may be required for elderly patients with dementia.

We selected the article that targeted subject with dementia and the assessment tools of the cognition that the reliability and validity were proven were used in the paper. We decided the assessment of tool in the targeting paper was for subject with dementia and it was not associated with normal older people. Therefore, we did not consider the difference of cognition tool among the papers.

## 5. Conclusion

In this study, the effects of exercise programs were reviewed; however, the appropriate intensity of the exercises was not analyzed. The number of analyzed papers was most likely too small because the papers that included subjects without a diagnosis of dementia were excluded, many papers described the intervention program effects qualitatively, and many papers were nonrandomized controlled (RCT) studies or did not have control groups.

In this study, the effects of physical activities by type on patients with dementia were objectively meta-analyzed to suggest a treatment program guideline for such patients. The papers with control groups and those that conducted before-after measurements were selected to determine the effect sizes of physical activity programs. The physical activity for patients with dementia had an effect on the improvement of physical capacity. Among the physical activities for patients with dementia, combined exercise, consisting of multiple exercises, showed the largest effect size. Therefore, physical activity programs that include various activities are recommended when treatment for elderly patients with dementia is determined. However, taking into consideration the diversity of the papers that were reviewed in this study, this outcome must be carefully applied to clinical cases.

## Figures and Tables

**Table 1:** The result of the homogeneity test for the sampling.

Number of studies	*Q*-value	df	*p*	*I* ^2^
9	102.17	8	0	92.17

**Table 2:** Characteristics of included trials.

Study	Type of intervention	*N*	Primary/secondary outcome	Items of intervention	Frequency	Duration (h)
Cheng et al. [8]	Special physical activity	12	Psychology state/GDS	Mahjong, Tai Chi	3 (60 min)	24

Eggermont et al. [2]	Special physical activity	30	Cognitive function/Wechsler Memory Scale revised, RBMT, Eight-Word Test (recognition)	Hand movements	5 (30 min)	6
			Psychology state/GDS and SCL Anxiety			

Kwak et al. [16]	Combined exercise	15	Daily activities	Stretching, thera-band, swiss ball, wall bar, dumb bell	5 (60 min)	6
			Physical capacity/cardiopulmonary function (m), muscle strength (kg W), muscular endurance (time), flexibility (cm), balance (s), agility (s)]			
			Cognitive function/MMSE			

Rolland et al. [17]	Walking with combined exercise	56	Activities of daily living	Collective exercise program (walk, strength, balance, and flexibility training)	2 (60 min)	48
			Physical capacity/(6 min walk speed (m/s), Get up and go (test score)			
			Psychology state (NPI, MADRS)			

Sung et al. [18]	Special physical activity	18	Psychology state (occurrence of agitated behavior)	Music with movement intervention	2 (30 min)	4

Venturelli et al. [1]	Walking	11	Physical capacity/6 min walk (m)	Walked up and down hallway	4 (30 min)	24
			Activities of daily living/Barthel, cognitive function/MMSE			

Vreugdenhil et al. [19]	Walking with combined exercise	20	Cognitive function/MMSE, ADAS-Cog	Community-based home exercise program [brisk walking (30 min), strength, balance training] + usual treatment	7 (30 min)	16
			Physical capacity/functional reach test (cm), Time Up and Go (s), sit to stand (number), waist/hip ratio, body mass index (kg/m^2^)			
			Abilities of daily living/Barthel index of ADL, Instrumental ADL			
			Psychology state/GDS			

Yágüez et al. (2011) [20]	Combined exercise	15	Cognitive function/CANTAB-Expedio	The Brain Gym training (activate balance, stretching, circular movements)	1 (90 min)	6

Eggermont et al. [4]	Walking	51	Cognitive function/Wechsler Memory Scale, RBMT	Walking at a self-selected speed	5 (30 min)	6

GDS: Geriatric Depression Scale, RBMT: Rivermead Behavioral Memory Test, SCL Anxiety: Symptom Check List, ADL: activities of daily living disability score, NPI: Neuropsychiatric Inventory, MADRS: Montgomery-Asberg Depression Rating Scale, ADAS-Cog: Alzheimer's Disease Assessment Scale-Cognitive Subscale, CANTAB: The Cambridge Neuropsychological Test Automated Battery.

**Table 3:** The results of publication bias verification results.

	Studies trimmed	Point estimate	95% CI		*Q*-value
			Lower limit	Upper limit	
Observed values		0.71	0.42	0.99	102.17
Adjusted values	0	0.71	0.42	0.99	102.17

**Table 4:** Effect size according to improvement of the dementia symptom.

Group	Number of studies	Point estimate	95% CI	*p* value
Physical capacity	14	1.05	0.03–0.73	0
Ability of activity of daily living	5	0.73	0.23–1.23	0.004
Cognitive function	29	0.46	0.26–0.66	0
Psychological state	8	0.39	0.01–0.77	0.045

**Table 5:** Effect size according to the type of physical activity program.

Category	Number of studies	Point estimate	95% CI	*p* value
Combined	15	1.17	0.86–1.47	0
Walking	13	0.46	0.18–0.74	0.001
Special physical activity	13	0.44	0.16–0.72	0.002
Walking with other activities	15	0.41	0.16–0.67	0.002
